# Death against Doctors

**Published:** 1887-04-30

**Authors:** 


### DEATH AGAINST DOCTORS.

He is a rare apostle who practises his own faith: a rarer doctor who keeps himself in health. Death is no respecter of persons, and fears neither prescriptions nor physic-bottles. "Skin for skin," said the Devil four thousand years ago; "all that a man hath will he give for his life." "Yea," saith the modern doctor, "but I must slowly give up even life itself in order to maintain my position, and to support my family in decency." These things have an end. The inexorable law of necessity will tell upon the medical profession in due time. Three horses cannot be summered upon one acre of grass. A leg of mutton a day can never be divided among three lions so as to satisfy them all. Sooner or later, and indeed sooner, either all the lions will die of hunger, or one will find the means of getting more than his share, and the other two will die. The doctors differ in nowise from the lions in this respect. If they are too thick upon the ground some must and undoubtedly will go to the wall. It will not necessarily be the best who will survive. It will be either the strongest or the most cunning—probably the most cunning—for among the higher animals cunning generally prevails over strength, and often over virtue.

Dr. Paget Thurstan, writing to the *Standard*, records some grim facts: and that they are facts ten thousand medical slaves can abundantly testify. The Hospital is not given to groaning or to counselling men to haul down their flags at every broadside the universal enemy may pour in. But it is given to keeping its eyes open and trying to understand the world in which it finds itself, and the general prospects for to-morrow's breakfast. "The mean net income of a general practitioner is about two hundred a year," says Dr. Thurstan. Two hundred a year! and this is the average. What, then, must be the income of the lower strata if this be the average income of all classes of general practitioners? What can a doctor do with two hundred a year? He must live in a good house, and wear a good coat and trousers, or nobody will employ him. He must keep a servant or servants, and dress his wife and children decently. And he must eat; he cannot lunch off pills or dine off rhubarb powder, or even cinchona bark. He might, it is true, use vaseline or simple ointment instead of butter for his bread, but the economy would be very doubtful.

"There are doctors in London," says Dr. Thurstan, "who charge fourpence a visit," and he might have added, "the visit is probably very dear at the money." For a doctor who values his visit at fourpence, has manifestly descended to the level of the crossing-sweeper or street-beggar, who takes any sum which the varying temper of the public will choose to bestow upon him. Such a doctor would probably allow himself to be kicked for sixpence, and touch his hat and say "Thank you, Sir," when the operation was finished. "There are doctors who thankfully accept one shilling a visit from patients living two miles from their doors." These are surely cases in which thankfulness is base and ingratitude would be meritorious. Finally, Dr. Thurstan says, "I am told that in one year, in which six hundred doctors died, about one thousand eight hundred medical students were entered to fill their places."

And what then? Nothing—except that doctors are dying very fast. This does not matter one whit to the general public. It is entirely a doctor's affair. With the public it is simply a question of supply and demand. Some time ago the horses of a certain tramway company lasted four years each on an average, and then died. An outsider, who felt compassion for the poor animals, asked whether with diminished work they could not be made to live much longer and be useful? "Oh, yes," was the answer; "but it pays better to work them very hard for awhile, and when they are worked out and die, to buy new ones." Simply a matter of profit and loss; nothing more. It is so with doctors; so with dock labourers and seamstresses, and every other class in which the supply is greater than the demand. Political economy—the grim science, which, although it has been relegated to Saturn by one of the most distinguished of modern statesmen, still persists in returning and remaining with us and asserting its power not only to survive but to rule.

The truth is, then, that doctors are dying rapidly, not because they are worked to death, but because they are worried to death. Worry is the dread spectre that drives life away. In the doctor's house it does not make a companion of the bony skeleton in his cupboard. It sits at his table, follows him to his carriage, invades his bed-chamber when he has come in from an anxious night visit, assails him at Christmas and at Midsummer, and at term-day when the school-fees are due. When is he rid of it? Never! For even in his dreams the dread vision, distorted into a thousand shapes of alarm, is still there.

"The doctor drinks," it is sometimes said; and no wonder. For, add to the paucity of his income the almost overwhelming anxiety which many a dangerous case brings to him, and it will be seen that to some men such a life would be almost insupportable, unless the pipe or the glass afforded a little temporary relief. We are not palliating the habit of taking stimulants to drown care—far from it. It is madness: it is ruin! But still, with some medical men it is almost a choice between stimulants and despair. Imagine a man like the late Tom Hood or Carlyle imprisoned, "cabin'd, cribb'd, confin'd," within the miserable routine of a low-class general practice. Either of them would have gone mad if he could not have changed his calling.

Can anything be done? Undoubtedly! Diminish the supply. Let ten thousand doctors emigrate, and turn farmers, or gold-diggers, or tavern-waiters, or anything that will give them an honest living. That is one remedy. Stop the inflow of students. That is another. Let every doctor make himself an apostle of prohibition. Let him explain to all his patients that one of the surest roads to poverty, drudgery, and never-ceasing care is the medical profession. True, many people will not believe him, because he lives in a large house and keeps a carriage. But let him assure them that all is not gold that glitters; and that where one doctor keeps a carriage and lives in a large house, two have to walk, to dwell on the edge of wretched slums, and to work day and night, Sunday and week-day, all the year round, for the privilege of dying of old age at fifty, and leaving a family of helpless children for somebody else to bring up.

